# Measurement properties of the Nepali version of the Connor Davidson resilience scales in individuals with chronic pain

**DOI:** 10.1186/s12955-018-0884-0

**Published:** 2018-04-03

**Authors:** Saurab Sharma, Anupa Pathak, J. Haxby Abbott, Mark P. Jensen

**Affiliations:** 1 Department of Physiotherapy, Kathmandu University School of Medical Sciences, P.O. Box No.: 11008, Dhulikhel, Nepal; 2 Centre for Musculoskeletal Outcomes Research, Dunedin School of Medicine, University of Otago, Dunedin, New Zealand; 3 Department of Rehabilitation Medicine, University of Washington, Seattle, USA

**Keywords:** Resilience, Clinimetric, Factor analysis, Reliability, Validity, Concurrent validity, Pain catastrophizing, Depression, Anxiety, Musculoskeletal pain, Psychometrics

## Abstract

**Background:**

Resilience is an individual’s ability to recover or “bounce back” from stressful events. It is commonly identified as a protective factor against psychological dysfunctions in wide range of clinical conditions including chronic pain. Resilience is commonly assessed using the Connor Davidson Resilience Scale (CD-RISC). Translation and cross-cultural adaptation of the CD-RISC into Nepali will allow for a deeper understanding of resilience as an important domain in health in Nepal, and will allow for cross-cultural comparison with other cultures. Therefore, the aims of the study were to translate and culturally adapt 10- and 2-item versions of the CD-RISC into Nepali and evaluate their psychometric properties.

**Methods:**

After translating the measures, we performed exploratory and confirmatory factor analyses of the 10-item version in two independent samples (ns = 131 and 134) of individuals with chronic pain. We then evaluated the internal consistency, test-retest stability, and construct validity of the 10- and 2-item measures in these samples. We also evaluated the internal consistency, and the construct and concurrent validity of the 2-item version in an additional sample of 140 individuals.

**Results:**

The results supported a single factor model for the 10-item measure; this measure also evidenced good to excellent internal consistency and excellent test-retest stability. Construct validity was supported via moderate associations with pain catastrophizing. The internal consistency of 2-item version was marginal, although construct validity was supported via weak to moderate associations with measures of pain catastrophizing, depression and anxiety, and concurrent validity was supported by strong association with the 10-item CD-RISC scale.

**Conclusion:**

The findings support the reliability and validity of the 10-item Nepali version of the CD-RISC, and use of the 2-item version in survey studies in individuals with chronic pain. The availability of these translated measures will allow for cross-cultural comparisons of resilience in samples of individuals with chronic pain.

## Background

Resilience has been defined as an individual’s ability to recover or “bounce back” from stressful events [1]. Resilience is gaining popularity as a domain for understanding variability in adjustment to chronic pain. It has been shown to be associated with physical disability and psychological function, including catastrophizing, anxiety and depression [1–3]. Those with higher resilience scores are more able to recover from painful states and use fewer analgesics than those with lower resilience scores [4, 5]. Resilience is commonly assessed in chronic pain research using the Connor Davidson Resilience Scale (CD-RISC).

The CD-RISC has three versions: the full 25-item [6], abbreviated 10-item (CD-RISC-10) [7] and brief 2-item (CD-RISC-2) [8] versions. The CD-RISC-10 was derived using exploratory and confirmatory factor analyses (EFA and CFA) and has been shown to have stronger validity than the full version [7]. Factor solutions of the original English version and translations have predominantly extracted one-factor solutions [7, 9–12]. The CD-RISC-10 has demonstrated good internal consistency [9, 11–13] and good to excellent test-retest stability over 2 to 6 weeks [9, 12, 13]. Validity has been supported via moderate associations with catastrophizing, anxiety and depression [12].

The CD-RISC-2 is made up of two items that are also in the CD-RISC-10. The CD-RISC-2 total score correlates strongly with the full-version of CD-RISC [8] and has previously shown acceptable internal consistency [8, 14] and good one-week test-retest reliability [15]. Its validity is supported via moderate associations with measures of depression and anxiety [14].

The CD-RISC-10 has been translated to many languages [10–12, 16–18], which facilitates cross-cultural comparisons. Translation and validation of the CD-RISC-10 and CD-RISC-2 into Nepalese will allow for a deeper understanding of resilience as an important domain in individuals in this unique population where the description of pain differs from populations from western cultures; for example, many more Nepalese than individuals from western cultures describe their pain using metaphors, and use pain descriptors which are difficult to translate into English [19].

The aims of this study were to develop and evaluate the measurement properties (psychometrics properties) of the 10- and 2-item Nepali versions of CD-RISC in individuals with chronic pain. We hypothesized that the Nepali CD-RISC-10 would demonstrate (1) a single-factor solution in two independent samples; (2) at least good internal consistency (Cronbach’s alpha ≥0.70 [9–13]); (3) good to excellent 2-week test-retest stability (*ICC* ≥ 0.70 e.g., [9, 12, 13]); and moderate negative correlations (i.e., *rs* ~ − 0.30) with pain catastrophizing [12]. We further hypothesized that the CD-RISC-2-NP would demonstrate (1) acceptable internal consistency, (2) good test-retest reliability [15], and (3) construct validity via moderate negative correlations (i.e., *rs* ~ − 0.30) with measures of anxiety and depression [12, 14]. We further hypothesized that the Nepali versions of CD-RISC-2 and CD-RISC-10 would (1) strongly correlate with each other, and (2) negatively correlate with the measure of pain intensity. Finally, we evaluated the standard error of measurement (SEM), minimum detectable change (MDC), and limits of agreement of both scales.

## Methods

We first translated the CD-RISC-10 and CD-RISC-2 into Nepali and evaluated the measurement properties of these scales in two independent samples of individuals with chronic pain.

### Translation procedures

Translation of CD-RISC-10 into Nepali was performed using standard patient-reported outcome measure translation guidelines [20]. After forward translations, synthesis of forward translations, and backward translation of the Nepali version of CD-RISC was completed, all the versions of the translations were evaluated by an expert committee, which consisted of the translators, researchers (SS, AP, MPJ), and Nepali language experts. After consensus among this expert committee members, a Nepali version of CD-RISC was finalised for pre-testing.

**Fig. 1:**
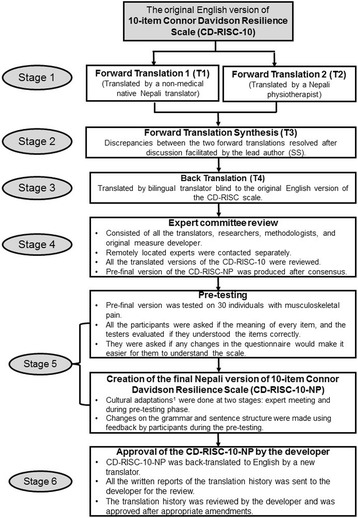
Translation history of Nepali CD-RISC-10

This Nepali version was pre-tested on 30 individuals with musculoskeletal pain selected to be representative of different ages, both sexes, and different education levels. After the completion of the Nepali CD-RISC, participants were asked to describe the meaning of each items. Feedback collected was used to improve the readability and ease of item understanding. The final version was reviewed and approved by Jonathan Davidson, one of the developers of the scale. Details of the translation history are presented in Fig. 1.

### Evaluation of measurement properties

The planned analyses and reporting of measurement properties was guided by COSMIN (COnsensus-based Standards for the selection of health Measurement INstruments) checklist [21, 22]. To evaluate the measurement properties of CD-RISC-NP, we used data from two samples. Ethical approval was obtained from the Institutional Review Committee of Kathmandu University School of Medical Sciences, Nepal (reference number 75/15). Written informed consent was obtained from every participant before the collection of data. Participant who were unable to read or write in Nepali provided verbal consent, and a witness signed the consent form on their behalf.

### Participants

To evaluate the measurement properties of CD-RISC-NP measures, we used the two samples that were also used to evaluate the psychometric properties of Nepali version of the Pain Catastrophizing Scale (PCS) [23]. However, although there is significant overlap in the participants between the previous study [23] and the current study, not the same participants completed both measures in both studies. The first sample in the current study consisted of 265 individuals with chronic pain recruited from a tertiary care hospital (*n* = 22) or the community (*n* = 243) in Nepal. This sample was further divided into two subgroups (*n* = 131 and *n* = 134) for the planned exploratory and confirmatory factor analyses (EFA and CFA) of the CD-RISC-10-NP, hereafter called the EFA sample and CFA sample, respectively. The CD-RISC-10-NP, the 7-point Global Rating of Change (GROC), the 3-item Patient-Reported Outcome Measurement Information System (PROMIS) pain intensity scale short-form 3a, and the 13-item PCS were administered to this sample. Two hundred and twenty nine (86%) of these individuals were re-administered the CD-RISC-10-NP after 2 weeks. Test-retest reliability was computed on a subgroup (*N* = 113) who endorsed “no change” on the GROC scale over this time period.

The second sample consisted of 140 individuals with chronic musculoskeletal pain recruited from community (*n* = 100) and the same tertiary hospital (*n* = 40). Nepali versions of 21-item Beck Depression Inventory (BDI), 21-item Beck Anxiety Inventory (BAI), and PCS were administered concurrently with CD-RISC-2 to this sample.

### Measures

#### Resilience

The Nepali version of 10-item and 2-item CD-RISC were administered to samples 1 and 2, respectively [8]. Each of the CD-RISC-10-NP items is rated on a 5-point Likert scale ranging from 0 = “*Not true at all*” to 4 = “*True nearly all the time.*” The two item scale sums to a possible high score of 8, and 10-item scale sums a possible high score of 40. Higher scores indicate more resilience. The two items included in the 2-item version were recommended by the developers as the two items that best represent the resilience construct; they were “I am able to adapt when changes occur” and “I tend to bounce back after illness, injury or other hardships” [15].

#### Depression

The Nepali version of the 21- item Beck Depression Inventory (BDI) was used to assess depression. With the BDI, respondents are asked to indicate the severity of depressive symptoms using 4-point scales ranging from 0 to 3. Each 4-point scale response is specific to individual items. Total scores can range from 0 to 63, with higher scores indicating more depression. The Nepali version of BDI has excellent psychometric properties (Cronbach’s alpha = 0.90 and two week test-retest reliability = 0.84) [24, 25]. The BDI evidenced excellent internal consistency (Cronbach’s alpha = 0.90) in the second chronic pain sample of the current study (*n* = 140).

#### Anxiety

The Nepali version of 21-item Beck Anxiety Inventory (BAI) was used to assess anxiety. With the BAI, participants are asked to rate each item on a 4-point Likert scale ranging from 0 (“*Not at all*”) to 3 (“*Severely, I could barely stand it*”), with possible total scores ranging from 0 to 63. Higher scores indicate greater anxiety. The Nepali version of BAI has shown to have excellent internal consistency (Cronbach’s alpha = 0.89) [26]. Internal consistency of BAI in the second chronic pain sample of the current study (*n* = 140) was good (Cronbach’s alpha = 0.89).

#### Catastrophizing

Catastrophizing was measured using a Nepali version of the 13-item Pain Catastrophizing Scale (PCS). Participants are asked to rate the frequency of their catastrophizing thoughts using a 5-point Likert scale ranging from 0 (“*Not at all*”) to 4 (“*All the time*”) [27]. Higher scores indicate higher catastrophizing. The Nepali version of PCS has been found to have excellent internal consistency (Cronbach’s alpha = 0.90–0.93) and test-retest stability (ICC = 0.90) [23]. In the current samples, which as indicated previously, largely but not completely overlap with the those used to validate the PCS-NP [23], the PCS evidenced excellent internal consistencies (Cronbach’s alphas = 0.91 and 0.93, in the first (*n* = 265) and second (*n* = 140) chronic pain samples, respectively).

#### Pain intensity

A Nepali version of the PROMIS pain intensity version 1.0 short form 3a scale was used to assess the pain intensity over the past week. It asks three questions regarding pain intensity: current pain intensity, worst pain, and average pain. Each item is scored on a 5-point Likert scale from 1 (“Had no pain”) to 5 (“Very severe”) [28]. A T-score representing characteristic pain intensity was calculated using response pattern scoring as recommended by PROMIS [28].

#### Global rating of change

A Nepali version of the Global Rating of Change (GRoC) scale was used to assess the global rating of change in chronic pain-related problems in the sample 1 [29, 30]. It is a 7-point Likert scale ranging from 1 to 7. The mid-point 4 represents “No change”; higher scores indicate improvement and lower scores indicate worsening. The GRoC score = 4 was used to categorize the participants as “stable” or unchanged, and those who scored  > 5 as “Improved,” similar to previous studies [23, 29, 31]. We considered a one-point change as significant improvement in the GRoC scores [29–31]. The GRoC classification was used to help interpret a number of psychometric properties of the CD-RISC-10-NP and CD-RISC-2-NP (i.e., test-retest stability, SEM, MDC, and limits of agreement statistics). That is, we limited analyses for computing these statistics to those participants who reported no change in their GRoC scores (i.e., GRoC = 4) [32].

### Data analysis

All the data were analysed using SPSS version 24 except confirmatory factor analysis which was performed in AMOS for SPSS version 24. As indicated previously, the reporting of the measurement properties was guided by COSMIN recommendations [21].

#### Sample description

Descriptive statistics for the demographic variables (means and standard deviations for continuous variables, numbers and percentages for categorical variables) were computed to describe the sample.

#### Factor analyses

We first performed an exploratory factor analysis (EFA) using maximum likelihood as the method of factor extraction, and factor rotation was performed using Direct Oblimin (delta = 0) allowing factors to correlate with each other in the EFA sample. We then performed a series of confirmatory factor analyses (CFAs) in the CFA sample. Model fit was evaluated using the chi-square goodness-of-fit index, the ratio of chi-square value to degree of freedom, the root mean square error of approximation (RMSEA), comparative fit index (CFI), and parsimony goodness-of-fit index (PGFI). We determined that a model had a good fit if (1) the chi-square value and the ratio of chi-square to degree-of-freedom values were relatively close to zero, (2) the RMSEA value was low and close to 0, (3) the CFI was large and close to 1, and (4) the PGFI value was large and close to 1 [33].

#### Reliability

We computed internal consistencies for both versions of CDRISC-NP scales; using Cronbach’s alpha for CD-RISC-10-NP and Spearman-Brown Coefficient for the CD-RISC-2-NP [34]. We considered internal consistencies between 0.70 and 0.79 as adequate, 0.80 and 0.89 as good, and values 0.90 or larger as excellent [35]. Two-week test-retest stability was evaluated using the intraclass correlation coefficient (ICC) in the stable group who endorsed no change in response to the GRoC. We considered ICC values of .75 or more as excellent [35].

SEM is another reliability parameter recommended by COSMIN checklist to describe measurement error which compliments temporal stability of a scale [22]. Larger scores of SEM indicate large variability and indicate more error, and smaller scores indicate minimal variability and suggest high precision. We calculated the SEM using the formula, SEM = SD_change_ x √(1 - ICC) [32] where SD_change_ is the standard deviation for the mean change score of CD-RISC-NP. We then computed MDC_95%_ for the CD-RISC-NP scales using the formula, MDC_95%_ = 1.96 x √2 x SEM [22, 32]. Finally, we created two Bland-Altman plots to indicate the levels of agreement of CD-RISC-NP scorings between the baseline and follow-up assessments for CDRISC-2-NP and CDRISC-10-NP separately [21, 36]. The plot was drawn using change in CDRISC-NP scores between baseline and follow-up in the Y-axis, and mean score of CDRISC-NP between baseline and follow-up assessments in the X-axis.

#### Validity

We evaluated the construct validity of the CDRISC-NP scales by computing the correlations of the baseline data of CD-RISC-NP scales with the baseline scores of Nepali versions of BDI, BAI, PCS administered in the EFA sample and PROMIS-PI administered to CFA sample, using Pearson correlation coefficients.

We also performed concurrent validity of CD-RISC-2-NP scale by evaluating its correlation with CD-RISC-10-NP, and hypothesized that CD-RISC-2-NP to have concurrent validity if the correlation coefficient was 0.70 or more [37].

## Results

### Translation of CD-RISC-10 into Nepali

The Nepali version of CD-RISC-10 was easy to understand, and retained its original meaning. Cultural adaptations were made on three of the CD-RISC-10 items (i.e., items, 1, 3, and 4). The cultural adaptations are reported in Appendix 1.

### CD-RISC scores

The CD-RISC-2-NP and CD-RISC-10-NP scores were normally distributed in all the three samples at all assessment points. The means and SDs of the CD-RISC-2-NP and CD-RISC-10-NP scores are presented in Table 1. A total of 83% (*n* = 109/131) and 81% (*n* = 106/131) in EFA sample and 95% (*n* = 128/134) and 92% (*n* = 123/134) completed follow-up assessments in the CFA sample for CD-RISC-2 and CD-RISC-10, respectively.

**Table 1:** Description of the study participants and the CD-RISC scores

	Sample 1		
	EFA sample	CFA sample	Sample 2
	(*N* = 131)	(*N* = 134)	(*N* = 140)
Variable	N (%) or Mean (SD)	N (%) or Mean (SD)	N (%) or Mean (SD)
Recruitment, N (%)			
Community	109 (83%)	134 (100%)	100 (71%)
Hospital	22 (17%)	0 (0%)	40 (29%)
Primary site of pain, N (%)			
Multiple sites	60 (46%)	65 (48%)	60 (43%)
Low back and pelvis	21 (16%)	25 (19%)	31 (22%)
Knee	25 (19%)	28 (21%)	29 (21%)
Headache	6 (5%)	5 (4%)	0 (0%)
Neck	4 (3%)	0 (0%)	3 (2%)
Upper back	4 (3%)	0 (0%)	3 (2%)
Elbow	3 (2%)	1 (1%)	1 (1%)
Ankle and foot	3 (2%)	4 (3%)	1 (1%)
Shoulder	1 (1%)	1 (1%)	9 (6%)
Other sites	4 (3%)	5 (3%)	3 (2%)
Duration of pain in months,			
Mean (SD)	41.67 (56.19)	61.56 (74.14)	52.09 (76.94)
Follow-up assessment epoch in days,			
Mean (SD)	10.62 (1.42)	10.27 (1.82)	–
Age in years, Mean (SD)	44.92 (17.20)	47.91 (13.81)	47.27 (14.54)
Sex, N (%)			
Men	37 (28%)	33 (25%)	50 (36%)
Women	94 (72%)	101 (75%)	90 (64%)
Religion, N (%)			
Hindu	123 (94%)	102 (76%)	130 (93%)
Buddhist	6 (5%)	27 (20%)	4 (3%)
Others	2 (1%)	5 (4%)	6 (4%)
Race/Ethnicity, N (%)			
Chettri	10 (8%)	6 (4%)	59 (42%)
Brahmin	71 (54%)	13 (10%)	38 (27%)
Newar	40 (30%)	101 (75%)	19 (14%)
Others	10 (8%)	14 (11%)	11 (8%)
Education, N (%)			
No school	25 (19%)	15 (11%)	44 (31%)
Primary school (<5 years)	32 (24%)	20 (15%)	42 (30%)
Secondary school (6-10 years)	23 (18%)	34 (25%)	34 (24%)
Higher secondary (11-12 years)	31 (24%)	25 (19%)	5 (4%)
Bachelor and over	20 (15%)	40 (30%)	15 (11%)
Primary occupation, ^a^ N (%)			
Unemployed	21 (16%)	0 (0%)	6 (4%)
Agriculture	37 (28%)	15 (11%)	48 (34%)
Homemaker	18 (14%)	36 (27%)	39 (28%)
Business	20 (15%)	37 (28%)	15 (11%)
Office worker	14 (11%)	24 (20%)	10 (7%)
Other	21 (16%)	22 (14%)	22 (16%)
CDRISC-2 score, Mean (SD)			
Initial assessment	5.21 (1.67)	5.47 (1.68)	5.43 (1.94)
Final assessment	5.20 (1.77)	5.54 (1.69)	–
CDRISC-10 score, Mean (SD)			
Initial assessment	27.05 (7.03)	28.54 (7.62)	–
Final assessment	26.25 (8.32)	27.72 (7.91)	–

^a^ As reported by the study participants

Total CD-RISC-2 score of 0/8 was reported by 0.4% of the participants in the sample 1 (*N* = 265; EFA and CFA sample combined), and 1% (*n* = 1) participants in the sample 2 (*N* = 140); and 8/8 score was reported by 10% (*n* = 26) of participants in the sample 1, and 16% (*n* = 23) of participants in the sample 2. Likewise, CD-RISC-10 score of 0/40 was reported by 0.4% (n = 1) and 5% (*n* = 13) of participants in sample 1.

### Handling missing items

In sample 1, there was missing item 3 CD-RISC-10 response for one participant, and a missing response to items 6 and 8 for another. There were no missing responses to the CD-RISC-2-NP items. Both the participants with missing items were excluded from all analyses involving the CD-RISC-10-NP.

### Demographic characteristics

The majority of the study participants were Hindu in religion (76% or more across samples) and were women (65% or more across samples). The plurality of participants (43% - 48% across samples) reported that they had chronic pain in more than one site. Of those reporting pain in one site, the most common sites were the low back and pelvis (16% - 22% across samples) and pain in the knees (19% - 21% across samples). Additional descriptive information for the study samples are presented in Table 1.

### Factor analyses results

**Fig. 2:**
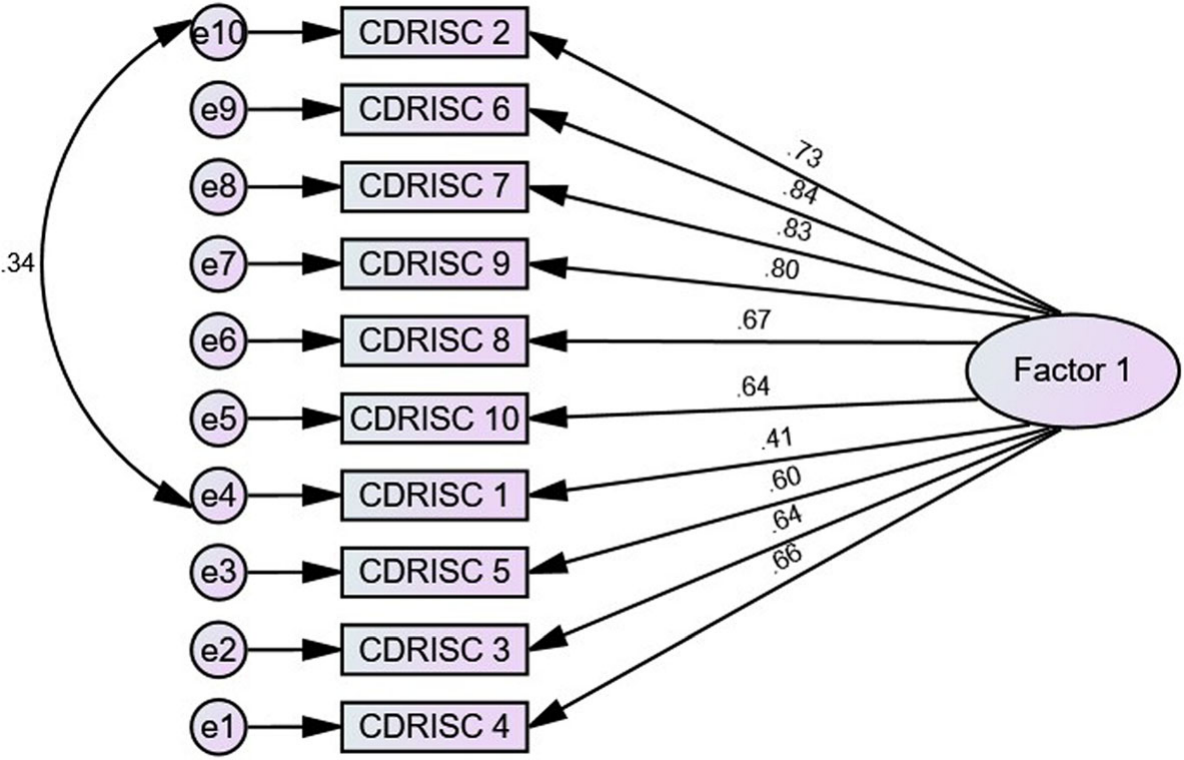
Path diagram after confirmatory factor analysis of CD-RISC-10 and covariance of error terms. Abbreviations: CDRISC, Connor Davidson Resilience Scale; CD-RISC-10, 10-item Connor Davidson Resilience Scale

The results of the EFA supported a single factor (first two eigenvalues were 4.98 and 0.90) for the CD-RISC-10-NP. This factor solution was confirmed via CFA in the CFA sample, which demonstrated a good fit. Covariance of error terms improved the fit index (Table 2). The path diagram of the CFA is presented in Fig. 2.

**Table 2:** Confirmatory factor analysis results for the 10-item Nepalese Connor Davidson Resilience Scale (CD-RISC-10-NP)

	*X*^*2*^ (df)	*X*^*2*^/df	RMSEA	CFI	PGFI
One-factor model	48.49 (35)	1.82	0.054	0.978	0.593
One-factor model with Modification^a^	34.33 (34)	1.01	0.009	0.999	0.588

^a^ Confirmatory factor analysis results after covariance of error terms e4 and e10

### Reliability results

The results of the reliability analyses for both the CD-RISC-10-NP and CD-RISC-2-NP are presented in Table 3. As can be seen, internal consistencies of CD-RISC-10-NP ranged between 0.87 and 0.90; and between 0.48 and 0.70 for CD-RISC-2-NP. The two-week test-retest reliability (Intraclass Correlation Coefficient) of CD-RISC-10-NP in the stable group (GRoC = 4) was 0.89; and 0.71 for the CD-RISC-2-NP. The SEM and MDC for both the 2-item and 10-item CD-RISC are presented in Table 3.

**Table 3:** Reliability of the Nepali versions of the 10- and 2-item Connor Davidson Resilience Scales

			Test-retest		
Sample	N	IC	ICC (95% CI)	SEM	MDC_95%_
CD-RISC-10					
Total sample	265	.89			
EFA sample	131	.88			
CFA sample	134	.87			
Stable group	113	.90	.89 (.86, .92)	2.42	6.72
CD-RISC-2					
Total sample	265	.55			
EFA sample	131	.62			
CFA sample	134	.48			
Stable group	119	.70	.71 (.58, .80)	0.86	2.38
Sample 3	140	.60			

*CD-RISC-10* 10-item Connor Davidson Resilience Scale, *CD-RISC-2* 2-item Connor Davidson Resilience Scale, *IC* Internal consistency (Cronbach’s alpha for the CD-RISC-10 and Spearman-Brown correlation coefficient for the CD-RISC-2); *SEM* Standard Error of Measurement (SEM = SD_change_ x √(1-ICC)); MDC_95%_, Minimum Detectable Change for 95% Confidence Interval (MDC_95%_ = Z x √2 x SEM); *ICC* Intraclass Correlation Coefficient

**Fig. 3:**
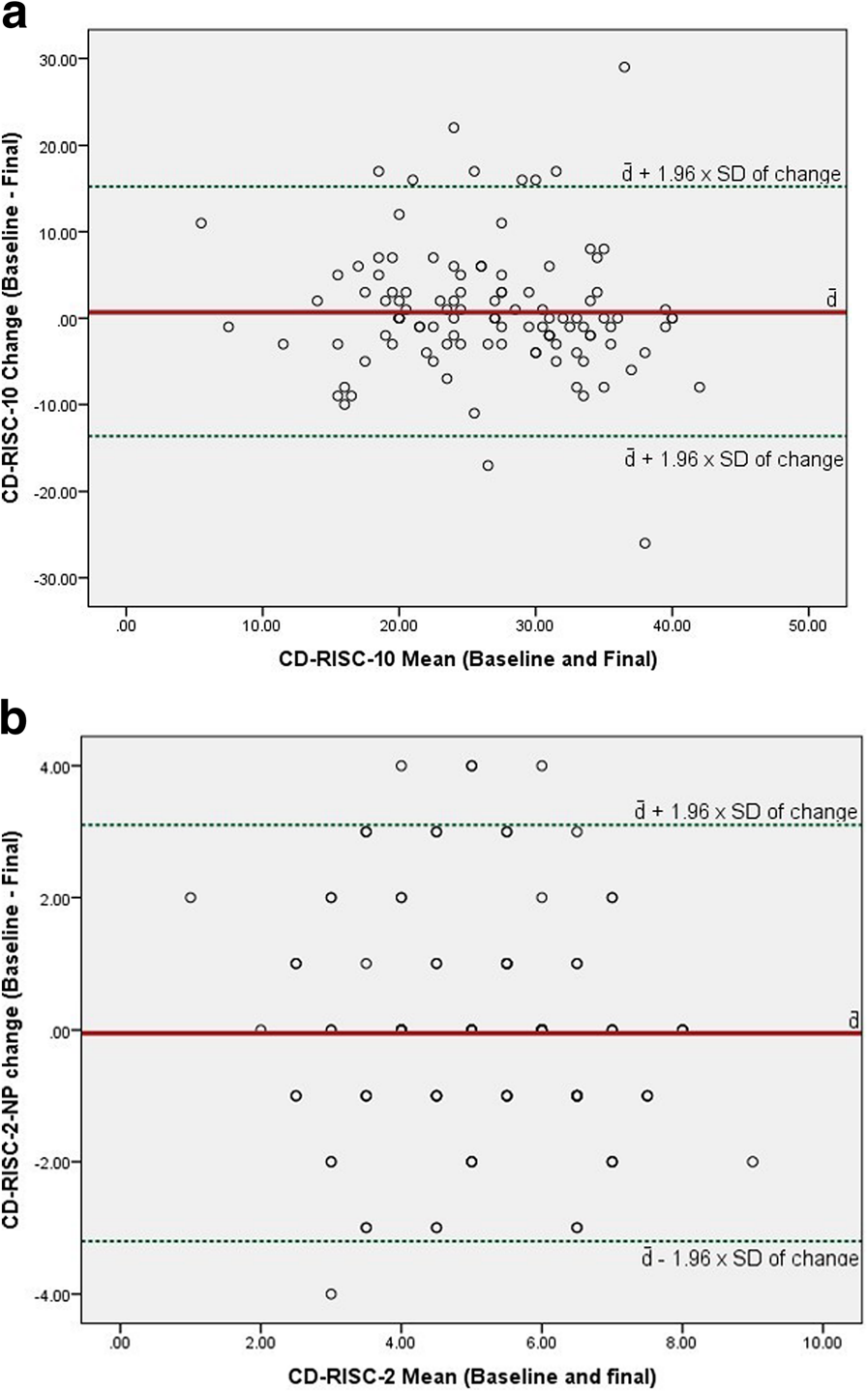
Bland-Altman Plot for CD-RISC-10 (**a**) and CD-RISC-2 (**b**). Note: Y-axis is the change of CD-RISC scores between baseline and follow-up measurements and X-axis is the mean of the CD-RISC scores at the baseline and final measurement. Solid line is the mean change of score (***d̄***); and dotted lines are *d̄* ± Z x SD_change_ (where Z = 1.96 for 95% confidence interval)

The Bland-Altman Plots demonstrating the limits of agreement are presented in Fig. 3. Figure 3a and b show graphical representations of the systematic and random errors of test-retest measurement scores for the 10-item and 2-item CD-RISC assessed in the stable group, respectively. The central red lines represent the systematic error, and the green dotted lines represent random errors of test-retest scores.

### Validity results

Construct validity of CD-RISC-10-NP was supported by significant moderate negative correlations with the PCS, (*rs* = .30–.45, Ps < 0.001); the CD-RISC-2-NP showed weak to moderate associations with the PCS (*rs* = .23–.35, *P* < 0.001). Validity of CD-RISC-2-NP was supported by a moderate negative correlation with depression and weak and negative association with anxiety. Finally, CD-RISC-2-NP and CD-RISC-10-NP were negatively (weakly to moderately) associated with the measure of pain intensity. The CD-RISC-2-NP demonstrated concurrent validity by strong positive association (*r* = 0.75) with its longer version, CD-RISC-10-NP. The construct validity of the CD-RISC-NP scales with their correlation coefficients with comparator instruments are presented in Table 4.

**Table 4:** Construct validity (*r*) of the Nepali versions of the 10- and 2-item Connor Davidson Resilience Scales

Sample	N	PCS	BDI	BAI	PROMIS
CD-RISC-10					
Total sample	265	−.35**			−.27**
EFA sample	131	−.45**			−.37**
CFA sample	134	−.30**			−.23**
Stable group	113	−.36**			−.19**
CD-RISC-2					
Total sample	265	−.30**			−.27**
EFA sample	131	−.35**			−.38**
CFA sample	134	−.23**			−.17*
Stable group	119	−.24**			−.23**
Sample 3	140	−.43**	−.31**	−.27**	

*P < .05; **P < .01

*CD-RISC-10* 10-item Connor Davidson Resilience Scale, *CD-RISC-2* 2-item Connor, Davidson Resilience Scale, *BDI* Beck Depression Inventory, *BAI* Beck Anxiety Inventory, *NRS* Numerical Pain Rating Scale, *PROMIS* PROMIS pain intensity scale short form 3a

## Discussion

The translation and cross-cultural adaptation of CD-RISC-10-NP yielded a comprehensible, reliable and valid Nepali version consistent with the study hypotheses.

### Factor analyses

The findings support a single factor solution for the CD-RISC-10-NP, consistent with both the original English version [7] and translations [9–12] of this scale. To our knowledge, only the Nigerian version of the CD-RISC-10 [16] has shown a two factor solution, albeit with a very strong correlation between the two factors (*r* = 0.82).

### Reliability

The internal consistency of the CD-RISC-10-NP in our sample was similar to those previously reported [7, 10–12]. However, the internal consistency of the brief version (CD-RISC-2-NP) here was lower than those previously reported [8, 14]. The internal consistencies of CD-RISC-2-NP tends to be lower than that of CD-RISC-10-NP across all populations [7, 8, 11, 12, 14]. This could be due, in part, to the strategy chosen to develop the CD-RISC-2. That is, the two items of the CD-RISC-2 were chosen from the original 25 CD-RISC items to capture the view of the meaning of resilience, without the guidance of empirical tests [15]. Use of such tests, including Item Response Theory or Rasch analyses, could potentially yield a brief version of the CD-RISC that has greater reliability. Additional work to develop alternative brief versions of the CD-RISC with greater reliability appears warranted.

We found a very high 2-week test-retest stability for the CD-RISC-10-NP, similar to the 6- and 2-week test-retest stability coefficients previously reported in two studies [9, 12], and higher than (but still adequate) 2-week test-retest stability coefficient (0.71) reported in a third study [13]. In the current study, we assessed test-retest stability only in a group of participants who reported no global changes in pain-related problems (i.e., a stable group). This is a recommended method for the reporting of the temporal stability of a patient-reported outcome measures [21, 32]. Use of a stable group is important, because resilience scores could potentially change after an intervention [15] or after facing adversities in life.

Our study presents novel findings regarding the measurement errors (SEM and MDC) of the CD-RISC scales. It is important to consider both of these when interpreting results of a measure to be used in longitudinal research, because not every scale score change represents a true (reliable) change. The MDC is the amount of change beyond measurement error, and thus represents the amount of change that can be considered reliable. Our findings suggest that changes in the CD-RISC-10-NP and CD-RISC-2-NP of 6.72 (scale 0–40) and 2.38 (scale 0–8) represent true changes; values below these are more likely to be due to measurement errors than values above these cutoffs. The Bland-Altman Plot [36] results (Fig. 3) provides visual information regarding the limits of agreement; that is, how far the retest scores deviate from the test scores, indicating general agreement between the two assessment points.

### Validity

Both the 2-item and 10-item CD-RISC-NP demonstrated construct validity via moderate associations with measures of pain catastrophizing and negative associations (weak to moderate) with pain intensity. The validity of the 2-item scale was supported via its moderate negative associations with measures of pain catastrophizing and depression, and weak but still significant association with measure of anxiety. These findings are consistent with previous research, and support the validity of the 10-item Nepalese versions of the scales [12, 14]. Less, but still adequate, support for the validity of the CD-RISC-2-NP was found, via its weak association with anxiety in our sample. To our knowledge only one study has previously evaluated the association of CD-RISC-2 with anxiety, which showed a moderate correlation [14].

The CD-RISC-2-NP demonstrated its concurrent validity by a strong association with the CD-RISC-10-NP. The magnitude of association found here was similar to that found in a previous study (*r* = 0.77; [14]), although it was somewhat lower that that found in another study (*r* = 0.88; [8]).

### Strengths and limitations

An important strength of the current study is that we followed the standard translation guidelines for the translation and cross-cultural adaptation of health-related patient reported measures [20]. We also followed COSMIN recommendations for the reporting of measurement properties of the 2-item and 10-item CD-RISC-NP scales [21, 22], which is the current reference standard for reporting measurement properties. The psychometric properties of CD-RISC-NP measures were tested in three different samples (including the analyses for factor structure), with Ns > 100 for each sample, which is minimum recommended for the assessment of psychometric properties. Test-retest stability was also assessed in more than 100 participants (as recommended by COSMIN [21, 22]) who reported little change in their pain problem, as assessed by the GRoC [32]. Finally, we also evaluated the SEM and MDC of CD-RISC-NP, which we believe is the first time these important statistics have been reported for the CD-RISC-2 and CD-RISC-10 measures.

Although the study has important strengths in terms of sample size, methodology, and rigour, the study’s limitations should also be considered when interpreting the findings. One limitation is that the back translation of the measure was performed by a single back-translator; translation and cross-cultural adaptation guidelines described by Beaton and colleagues [20] recommend that two or more translators to perform the back translations. This weakness might be mitigated some by the fact that translation guidelines indicate that use of a single back translator is acceptable [38]. As the items of the CD-RISC are fairly straightforward, translation was relatively simple and we found few issues during the cognitive testing of the items. Moreover, the few issues that emerged were easy to resolve (see Appendix 1). The adequacy of this approach was also supported by the strong psychometric properties of the resulting scales. Still, use of two or more back translators would have been ideal.

A second important limitation of the study is that we used a GRoC scale that asked participants to rate global change in pain-related problems to categorize participants as “unchanged” in order to evaluate test-retest stability of the CD-RISC-NP scales. Although, asking participants to rate their change in “resilience” would have been ideal to categorize participants into stable or improved groups, using "global" scores to assess test-retest stability is a common practice (e.g., [32, 39, 40]). The excellent reliability scores in the present study are consistent with idea that the participants may have considered resilience into account when rating overall change. Future studies may evaluate relationships between patient’s “global rating of change” and “change in resilience” scores, to explore if they are related, or conduct temporal stability of resilience measures using change of the resilience scores instead of GRoC.

Third, the GRoC score asks participants to recall the change in pain-related problem since the baseline assessment, which may introduce a recall bias. However, the duration of reassessment of only approximately 10 days likely limited recall bias, and is less than the recommended duration of 2 weeks for the assessment of test-retest stability [21].

Fourth, we used cross-sectional data to assess the validity of the resilience measures; such data do not allow us to draw causal inferences regarding the associations among the domains assessed by the study measures. Future research is needed to evaluate the causal relationship between resilience and psychological functions in individuals with chronic pain from Nepal.

Fifth, we assessed the measurement properties of CD-RISC-NP in adult Nepalese with chronic pain only. The findings therefore do not necessarily generalize to other populations or who have other clinical conditions, for example paediatric populations and those with mental health conditions. Future research is required to evaluate the validity of CD-RISC-NP measures in these populations.

Finally, we did not evaluate the responsiveness of the CD-RISC-NP measures to treatment. It would have been ideal to evaluate the minimum important change (MIC) score of the CD-RISC-NP, which could be used as reference to evaluate clinical meaningful improvement. Future studies may evaluate responsiveness and MIC to determine the utility of the CD-RISC-NP scales as outcome measures would be useful.

## Conclusions

In summary, the 10-item CD-RISC-NP scales evidenced good measurement properties; the findings support the use of this measure in research studying resilience in chronic pain populations. The results provide less support for the reliability and validity of the 2-item CD-RISC-NP, but indicate that it could be used in studies using larger samples (e.g., survey studies). Research is needed to better understand the causal influence of resilience on psychological function, and how this might differ as a function of language and culture.

## Appendix 1

### Key cross-cultural adaptation of the Nepali CD-RISC-10 items

- **Item 1:** Item 1 was originally translated as “कुनै परिवर्तन हुँदा म परिस्थिति अनुसार ढल्न सक्छु”. The original Nepali word used in this item for “adapt” was “ढल्न”. However, during the pre-testing for first few participants, patients misunderstood this word as “fall” or “faint” which are other meanings of this word. Thus, during the pre-testing phase, we added “परिवर्तन हुन” within parenthesis which translated to “change” to clarify meaning of “ढल्न” as “adapt” or “change.” After making this change, remaining majority of participants witnessed further issues with this item during pre-testing after this change was made.
- **Item 3**: Item 3 was originally translated as “जब म कुनै समस्याजनक परिस्थितिमा हुन्छु, म त्यसका हास्यास्पद पक्षहरू हेर्ने प्रयास गर्छु”. However, participants struggled to understand meaning of Nepali word for “humor”. We therefore added “रमाइला” to the item, which means “fun” within parenthesis of “हास्यास्पद” which clarified the meaning of the item. There were no further issues with this item during pre-testing after this change was made.
- **Item 4:** The forward translations of this item was translated as “तनावको सामना गर्नाले मेरो बल झन् दरो हुन्छ”. The use of the word “बल” which means strength in Nepali is ambiguous. Thus, in the expert committee meeting, we decided to replace the word by “मनोबल” which means “mental strength” to clarify its meaning in Nepali.

## Abbreviations

CD-RISC-10: 2-item Connor-Davidson Resilience Scale
CD-RISC-10-NP: Nepali version of 10-item Connor-Davidson Resilience Scale
CD-RISC-2: 2-item Connor-Davidson Resilience Scale
CD-RISC-2-NP: Nepali version of 2-item Connor-Davidson Resilience Scale
CI: Confidence Interval
COSMIN: COnsensus Based Standards for the selection of health Measurement INstruments
GROC: Global Rating of Change
GROC-NP: Nepali version of Global Rating of Change
ICC: Intraclass correlation coefficient
MDC: Minimum detectable change
MDC_95_: Minimum detectable change at 95% confidence margin
PROMIS: Patient-reported outcome measurement information system
SD: Standard deviation
SEM: Standard error of measurement
